# N-acetyl Cysteine Supplementation to Alleviate Skin Picking Disorder: A Case Report

**DOI:** 10.7759/cureus.53440

**Published:** 2024-02-02

**Authors:** Samira Khan, Sarah Hughes, Olivia Hill

**Affiliations:** 1 Behavioral Health, West Virginia University (WVU) - Berkeley Medical Center, Martinsburg, USA; 2 Behavioral Medicine and Psychiatry, West Virginia University School of Medicine, Martinsburg, USA

**Keywords:** vitamin, mood, pediatric, anxiety, n-acetylcysteine (nac)

## Abstract

There are body-focused repetitive behaviors, such as skin picking, trichotillomania, or nail biting, for which therapeutic interventions are available and can be tried, but unfortunately, there are no FDA-approved medications specifically for them. These disorders can cause functional impairment, disrupt activities of daily living, and be burdensome for both the patients and their loved ones. This case report will discuss an over-the-counter vitamin supplement, N-acetyl cysteine (NAC), that can be used safely but is often overlooked.

## Introduction

Skin-picking disorder (SPD), also referred to as excoriation disorder or dermatillomania, is a body-focused repetitive behavior where an individual excessively picks at their own skin to the point of having open wounds that do not heal properly and can lead to skin infections. SPD can be comorbid with other mental health disorders like anxiety or obsessive-compulsive disorder (OCD). Interventions such as cognitive-behavioral therapy (CBT), involving habit reversal training and/or stimulus control, are the first-line treatment followed by medications, such as selective serotonin reuptake inhibitors (SSRI), which treat the underlying mental health issues [1,2]. N-acetyl cysteine (NAC) is an amino acid derivative generally known for its use after an acetaminophen overdose and as a mucolytic [2]. There is growing evidence for its use as a treatment for psychiatric disorders related to impulse control, such as skin picking, trichotillomania, OCD, autism, and substance abuse. NAC’s beneficial properties may derive from its role in neurogenesis, apoptosis, oxidative stress, neuroinflammation, and the regulation of dopamine and glutamate [2].

## Case presentation

The patient is a 13-year-old boy, who was initially evaluated in 2021 at the age of 11 by an outpatient child and adolescent psychiatrist for anxiety, Attention deficit hyperactivity disorder (ADHD), and OCD symptomatology. On presentation, his anxiety presented as difficulty with and fear of meeting new people and initiating conversation. His OCD symptomatology included requiring his room to be organized in a particular way and physical and emotional outbursts if it was not. Additional OCD behavior included watching his food being made and his subsequent refusal to eat it if he was dissatisfied with its preparation. The patient also experienced sensory sensitivity including discomfort with specific clothing textures resulting in him sleeping directly on the mattress, due to his dislike of the sheet’s consistency.

After the first visit, his physician prescribed Prozac 20 mg and Adderall 10 mg for his anxiety, OCD, and ADHD symptoms, which initially improved. However, on his next follow-up visit, his father noted that the patient recently began picking at his skin, leading to open wounds on his hands, arms, and legs. Following this revelation, he was started on 600 mg capsules of NAC to treat his excoriation tendencies. The parents were advised to increase the dosage to 1200 mg after four weeks and to continue that dose and monitor him until the next visit.

At his two-month follow-up, he continued to experience similar symptoms with no change in his skin-picking compulsion after taking the 1200 mg of NAC. In addition to increasing his Prozac to 30 mg, NAC was increased to 1800 mg capsules. Due to non-compliance with his appointments, the patient was lost to follow-up for approximately 11 months. Nevertheless, when he returned for his visit with the same concerns, NAC was increased to 2400 mg. At each subsequent follow-up visit, the family reported improvement in the patient's skin-picking, and so the patient remained on that dose. A year later, his parents noted that the patient only exhibited skin-picking when he did not receive his NAC supplement, so they have continued to provide that to him consistently. Today, the patient continues to remain on this stable dose, NAC 2400 mg once a day, due to it effectively eliminating his SPD.

## Discussion

Though the existing body of evidence is fairly small, the patient’s response is consistent with the current literature regarding NAC as a treatment for SPD. In one study, all 35 subjects reported improvement in SPD following the treatment of 450-1200 mg NAC [2]. Another showed a significant reduction with 1200-3000 mg per day, and multiple case studies have shown significant improvements with 1200-1800 mg per day [3]. Hair-picking and nail-biting disorders have also shown significant improvement with NAC supplementation in the literature, supporting the influence of NAC on impulse control disorders as a whole [4]. Excess glutamate is thought to be responsible for difficulty with impulse control, so one goal of this spectrum of psychiatric disorders is reducing glutamate release. The hydrolysis of NAC yields cysteine, which is converted to cystine in the brain and exchanged for glutamate, thereby inhibiting further glutamate release within the nucleus accumbens [2]. Furthermore, excess glutamate can damage excitatory neurons by stimulating N-methyl-D-aspartate (NMDA), leading to excitatory neurotransmission dysregulation [5]. On the spectrum of impulse control disorders, patients with OCD have also shown much higher levels of glutamate in their cerebrospinal fluid (CSF) than controls [4]. One study’s magnetic resonance spectroscopy results showed that NAC can restore glutamate balance in those with cocaine use disorder, supporting the proposed relationship of NAC, glutamate, and impulse control [5]. Increased glutamate results in increased glutamine, which contributes to dopaminergic signaling inhibition; this could contribute to reduced baseline dopamine in those with SPD and drive them to perform compulsions to seek reward [6]. Glutamate dysregulation can also reduce the ability of brain neurons to appropriately adapt to their amount of stimuli, known as long-term potentiation and depression (LTP, LTD), and neuroplasticity. One study in rats showed that rats with damaged LTP and LTD due to cocaine use disorder were able to redevelop LTP and LTD following NAC treatment [5]. The ability to adapt to frustrating situations involves LTP and LTD, which is likely dysregulated in those with impulse control disorders, making neuroplasticity a promising therapeutic target for future studies. While a more general mechanism, oxidative stress could be a potential factor in the pathophysiology of SPDs, as many other psychiatric disorders can be attributed to oxidative stress, including OCD. Cell death and inflammation can result in neurotransmitter dysregulation and dysfunction, which may result in an aberrant glutamate balance and reduced impulse control. NAC is an antioxidant, reducing superoxide radicals and mitigating oxidative stress and damage to brain neurons, which likely explains NAC’s symptomatic reduction in SPD [5]. While more research is needed on the specific oxidative damage involved in impulse control disorders such as SPD, NAC’s antioxidant properties propose a promising therapeutic option.

## Conclusions

Impulse control disorders can cause significant distress and disruption to daily life, and existing pharmacological therapies are limited. The amino acid derivative NAC has shown promise as a potential treatment by regulating glutamate concentration, reducing oxidative stress, and influencing dopamine metabolism. This case study of a 13-year-old male with SPD, successfully treated and resolved with 2400 mg of NAC, contributes to the existing evidence literature regarding NAC's potential therapeutic role in impulse control disorders. Larger clinical trials are needed to further establish NAC's efficacy.
